# Effectiveness of a 3-Month Mobile Phone–Based Behavior Change Program on Active Transportation and Physical Activity in Adults: Randomized Controlled Trial

**DOI:** 10.2196/18531

**Published:** 2020-06-08

**Authors:** Anna Ek, Christina Alexandrou, Emmie Söderström, Patrick Bergman, Christine Delisle Nyström, Artur Direito, Ulf Eriksson, Pontus Henriksson, Ralph Maddison, Ylva Trolle Lagerros, Marcus Bendtsen, Marie Löf

**Affiliations:** 1 Department of Clinical Science Intervention and Technology Karolinska Institutet Huddinge Sweden; 2 Department of Health Medicine and Caring Sciences Linköping Sweden; 3 eHealth Institute Department of Medicine and Optometry Linnaeus University Kalmar Sweden; 4 Department of Biosciences and Nutrition Karolinska Institutet Huddinge Sweden; 5 Yong Loo Lin School of Medicine National University of Singapore Singapore Singapore; 6 Strömstad Academy Strömstad Sweden; 7 Institute for Physical Activity and Nutrition School of Exercise and Nutrition Sciences Deakin University Melbourne Australia; 8 Department of Medicine Clinical Epidemiology Unit Karolinska Institutet Stockholm Sweden; 9 Center for Obesity Academic Specialist Center Stockholm Health Services Stockholm Sweden

**Keywords:** behavior change, mobile phone intervention, physical activity, active transportation, mobile phone app, smartphone app

## Abstract

**Background:**

Active transportation (AT; ie, walking and cycling as a mode for transportation) has been associated with decreased morbidity and mortality; however, low-cost and scalable intervention programs are lacking.

**Objective:**

The goal of the research was to determine the effectiveness of a 3-month behavior change program delivered via a mobile phone app to promote AT (TravelVu Plus) on time spent in moderate-to-vigorous physical activity (MVPA).

**Methods:**

For this 2-arm parallel randomized controlled trial, we recruited a population-based sample of 254 adults from Stockholm County who were aged 20 to 65 years and had access to a smartphone. On completion of 1-week baseline measures, the 254 participants were randomized to either the control or intervention group (1:1 ratio). Both groups had access to the standard TravelVu app (Trivector AB) for monitoring their AT for 6 months. The intervention group also received a 3-month behavior change program to promote AT (TravelVu Plus app). Assessors of outcomes were blinded to group allocation. Outcomes were objectively measured MVPA at 3 (primary) and 6 months. Secondary outcomes were AT, attitudes toward AT, and health-related quality of life at 3 and 6 months.

**Results:**

No effect on MVPA was observed after 3 months (*P*=.29); however, at 6 months the intervention group had a greater improvement in MVPA than the controls (6.05 minutes per day [95% CI 0.36 to 11.74; *P*=.04]). A Bayesian analyses showed that there was a 98% probability that the intervention had any effect at 6 months, and a 63% probability that this effect was >5 minute MVPA per day.

**Conclusions:**

No effect on MVPA immediately after the intervention period (at 3 months) was observed; however, there was a delayed effect on MVPA (6 minutes per day) at 6 months, which corresponds to approximately 30% of the weekly MVPA recommendation. Our findings suggest that a behavior change program promoting AT delivered via an app may have a relevant effect on PA.

**Trial Registration:**

ClinicalTrials.gov NCT03086837; https://clinicaltrials.gov/ct2/show/NCT03086837

**International Registered Report Identifier (IRRID):**

RR2-10.1186/s12889-018-5658-4

## Introduction

Physical inactivity (little or no physical activity) is a major risk factor for noncommunicable diseases, including cardiovascular disease, type 2 diabetes, and premature death [1] and contributes to high health care costs in both high- and low-income countries [2]. Low-cost, scalable interventions aimed at increasing habitual physical activity (PA) at the population level are warranted. Active transportation (AT; ie, cycling and walking as mode of transportation) represents a key target since AT is easily accessible and enables regular PA on a daily basis [3]. A recent meta-analysis (23 prospective studies, n=531,333) concluded that AT was associated with decreased mortality and lower risks of cardiovascular disease and diabetes [4]. Also, randomized controlled trials (RCTs) support that AT by bicycle can improve health markers such as insulin sensitivity and cardiorespiratory fitness [5-7]. However, to date, behavioral interventions targeting AT to increase daily PA in adults are few, and trials have been of mixed quality, with considerable variation in sample characteristics, study duration, and outcomes [8-12]. Furthermore, population-based RCTs assessing the effects of AT on PA in healthy adults using objective measures for both AT and PA are lacking.

Mobile health (mHealth) interventions are increasingly being used to promote healthy lifestyle behaviors [13,14] and include the use of mobile apps [15], which offer potential to be delivered at scale. To the best of our knowledge, only one app has focused on promotion of AT. In that study, Bopp et al [12,16] evaluated whether a campaign with self-monitoring of AT (via an app) together with social media and marketing components could increase AT among students and employees at a university campus. Results showed an increase in the number of self-reported active trips by students. However, due to the multicomponent nature of the intervention [12], it was not possible to evaluate the effect of the app component alone. Also, it was a single group study and only included self-reported travel. Thus, further well-conducted RCTs evaluating whether an app can promote AT are warranted.

This paper reports the results of the Smart City Active Mobile Phone Intervention (SCAMPI) trial. The aim of this trial was to determine the effectiveness of a 3-month mobile phone–based behavior change program promoting AT on moderate-to-vigorous physical activity (MVPA) in Swedish adults [17]. The primary outcome was MVPA at 3 months, while secondary outcomes included MVPA at 6 months as well as time spent in AT, perceptions about AT, and health-related quality of life at 3 and 6 months.

## Methods

### Study Design

A 2-arm parallel design RCT was conducted between September 2017 and September 2018. The study was approved by the regional research ethics committee in Stockholm (January 11, 2017: 2016/2403-31 and June 30, 2017: 2017/1373-32) and registered at ClinicalTrials.gov [NCT03086837]. Details on the development of the app and design of the SCAMPI trial are published elsewhere [17]. The study is reported according to the Consolidated Standards of Reporting Trials of Electronic and Mobile Health Applications and onLine Telehealth (CONSORT-EHEALTH) statement [18].

### Participants and Procedures

**Figure 1:**
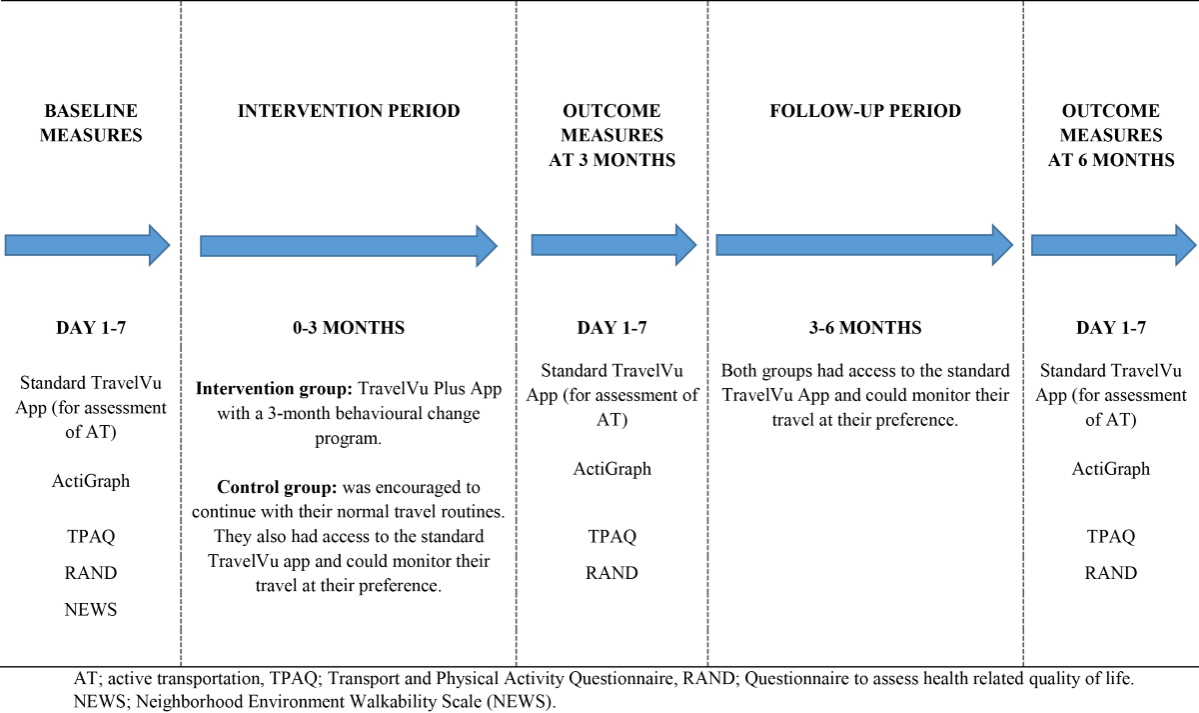
Description of the study design of the Smart City Active Mobile Phone Intervention trial.

Participants were recruited from a random sample of 4995 adults provided by Statistics Sweden. Two waves of invitation letters were sent out (September 2017 [n=2000] and January 2018 [n=2995]) in order to capture different seasons and weather. Participants were eligible if they were aged 20 to 65 years, understood written Swedish, lived in the county of Stockholm, and had access to a smartphone compatible with the app. Exclusion criteria was not being able to perform MVPA. People who wanted to participate in the study signed up at the study website or sent an email or a letter to the research group. After providing informed consent (electronically signed), a web-based questionnaire was administrated to collect self-reported sociodemographic variables (eg, age, sex, country of birth, educational attainment), height, weight, attitude toward AT, neighborhood walkability, and health-related quality of life. Thereafter, baseline measures of PA (using the wGT3X-BT accelerometer [ActiGraph LLC]) as well as AT (measured through the TravelVu app [Trivector AB], standard version) were assessed simultaneously during 7 consecutive days. The details of the TravelVu app have been provided in the study protocol [17]. Briefly, the app passively collected data using GPS coordinates and presented total and mode-specific travel minutes per day (for walking, cycling, car, train, ferry, or bus). At the end of each day during these 7 days, participants were asked to review and, if necessary, manually revise travel and locations in the app. After making the needed revisions to their travel routes, participants were instructed to approve these days by marking them as valid. All outcome measures were repeated postintervention at 3 months (primary time point) and 6 months postrandomization. An overview of the study design is provided in Figure 1.

### Sample Size, Randomization, and Blinding

A total of 250 participants (125 per group) was estimated to provide 80% power (α=.05) to detect a 10-minute difference in MVPA per day assuming a standard deviation of 25 minutes [19] and loss to follow-up of about 20%. On completion of baseline measures, participants were randomized to either the control or intervention group (1:1 ratio) using a computer-generated random allocation sequence list generated by the study statistician [17]. Allocation concealment was ascertained through opaque envelopes (ML). CA and ES enrolled participants. Assessors of outcomes were blinded; however, the group allocation was not blinded to the participants, who received an email after randomization.

### Control Group

After baseline measures, participants in the control condition were encouraged to continue with their normal travel routines during the 6-month study period. During this time they were able to continue to monitor their daily travels using the standard TravelVu app (without a behavior change program) if they chose to. At 3 and 6 months, participants in the control group (as well as in the intervention group) were asked to monitor their travel behavior in the app during the same seven days as they wore the accelerometer for follow-up outcome assessment.

### Intervention

In addition to the standard TravelVu app, intervention participants received a 3-month behavior change program (TravelVu Plus), aimed at increasing PA through AT. The program was delivered as extra features to the standard TravelVU app. The development of the TravelVu Plus program and its features are described in more detail in our study protocol [17]. Briefly, it was anchored in social cognitive theory [20] as well as social ecological principles [21] and included features such as a goal-setting function, messages (sent as push notifications), and feedback. In-app features encouraged participants to set new AT goals on a weekly basis. Feedback on participants’ AT and progress toward the set weekly goal were provided in graphical form throughout the week [17]. At the end of the week, push notifications were used to provide textual feedback on AT performance (ie, feedback on behavior) if PA goals were reached (ie, feedback on outcome of behavior) or to encourage modifying goal according to achievement (ie, review behavior goal). Furthermore, information on all achieved weekly goals thus far was provided graphically. Push notifications were also sent with general information on AT and its health and climate benefits as well as practical tips and behavior change strategies (Multimedia Appendix 1). After 3 months, the enhanced features were disabled and the intervention group had access to the standard version (ie, the TravelVu app) for the remaining study period (3 to 6 months after baseline) and could continue to monitor their AT. This enabled us to assess to what extent they used the self-monitoring of AT during the follow-up period and whether this was different from the control group.

### Outcomes

PA was measured objectively using the wGT3X-BT triaxial accelerometer (ActiGraph LLC), which was worn on the hip over seven consecutive 24-hour periods. Raw acceleration data (at 90 Hz) were uploaded and processed using the ActiLife software version 6.13.3 (ActiGraph LLC) into filtered sum of vector magnitudes (VM). Nonwear time was detected and excluded using a Troiano algorithm [22]. A day was categorized as valid if wear time ≥600 minutes [23]. For each participant, time spent in light PA (VM 201-2690 counts per minute), moderate PA (MPA; VM 2691-6166 counts per minute), vigorous PA (VM: ≥6167 counts per minute), and MVPA (VM ≥2691 counts per minute) were calculated using recommended cutoffs [23] by Sasaki et al [24], while time spent sedentary (VM 0-200 counts per minute) was derived by applying cutoffs by Aguilar-Farias et al [25].

To complement the accelerometer data, we also evaluated the number of minutes spent cycling and walking for transport assessed using the TravelVu app during seven consecutive 24-hour periods as a secondary outcome. Days marked as valid were used to assess mode and duration (minutes per day) of AT (cycling and walking). Days with unreasonably high levels of AT for the Stockholm area (>4 hours) were not included. Attitudes toward AT were assessed using the psychosocial items in section B of the validated Transport and Physical Activity Questionnaire [26]. Mean values for each AT mode (walking and cycling) were calculated for each participant. The RAND-36 was used to assess health-related quality of life [27,28]. The general health domain was analyzed in this study.

### Other Measures

Perceived neighborhood walkability was assessed at baseline using the Neighborhood Environment Walkability Scale questionnaire [29,30]. To calculate a perceived walkability index for each participant, we summed the z scores for residential density, street connectivity, and land use mix as described previously [19]. Finally, app engagement was measured as the number of registered days in the app (ie, days that the participant had reviewed and approved as valid regarding their travel behavior that day; intervention and control group) as well as the number of goals set and achieved (intervention group only).

### Statistical Analysis

All statistical analyses were conducted in accordance with the study protocol [17] and followed intention-to-treat principles. Linear mixed models (random intercept) were used to contrast differences in primary (MVPA) and secondary outcomes (AT, RAND-36 general health, attitudes toward using AT) between the intervention and control group. Outcomes were regressed against group allocation and included a group × time interaction term to incorporate repeated measures (0, 3, and 6 months).

Three-way interaction analyses were performed to assess if the following characteristics at baseline moderated the intervention effect on the primary outcome: sex, age, educational attainment, BMI, foreign background, season of randomization, perceived walkability index, attitude toward AT, or general health. We also examined whether the effect on MVPA was associated with engagement with the app (number of registered days as well as number of goals set and achieved in the app).

Due to relatively few missing values in the outcome measures (30/252, 11.9%, and 34/252, 13.5%, at 3- and 6-month follow-ups, respectively) and since we could not rule out the possibility that data were missing at random, we followed the recommendations to report completers only as the primary analyses [31-33]. A sensitivity analysis where missing data for the primary outcome at the 3- and 6-month follow-ups were imputed using multiple imputation with chained equations [33] (predictive mean matching, with 500 imputations and 30 iterations) was also conducted. Deviations from the missing completely at random assumption were evaluated through attrition analysis where baseline characteristics for completers and noncompleters were compared.

We also conducted the following analyses that were added to the statistical analysis plan before data analysis but not reported in the protocol [17]. First, since AT mainly corresponds to MPA, the largest component of MVPA, we also used linear mixed models to contrast MPA between the two groups. Furthermore, recent data indicate that even light PA may reduce premature mortality [34-36], and since we cannot exclude that some AT would be light PA, we also ran models with light PA as outcome. Additionally, we explored whether accelerometer wear time influenced our results; however, our estimates remained similar after adjustment (results not shown). Finally, in exploratory analyses, Bayesian inference for the linear mixed models was employed to calculate the probability that the intervention had an effect on MVPA [37]. These Bayesian analyses provided a more robust view of the data collected in the trial due to the following reasons: (1) *P* values and confidence intervals are not well defined in linear mixed models [38] and should therefore only be taken as approximate and (2) null hypothesis testing can be sensitive to individual data points [39]. Uniform priors were used for all parameters in the Bayesian analyses.

Statistical analyses were performed with a significance level of .05 using R version 3.6.1, and Bayesian inference was done using the probabilistic programming language Stan (RStan version 2.19.1, both R Foundation for Statistical Computing) and SPSS Statistics version 24 (IBM Corporation).

## Results

### Participants

**Figure 2:**
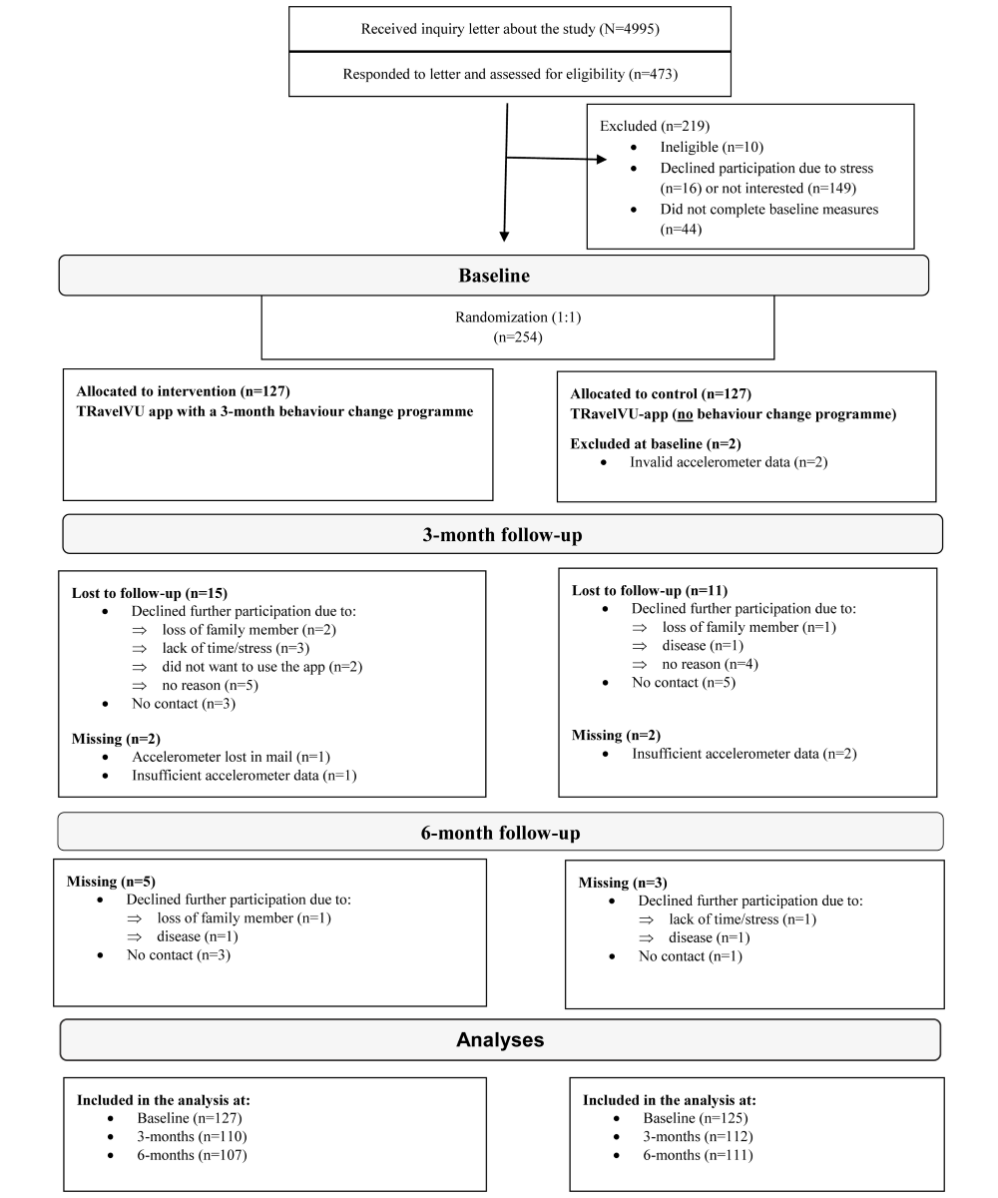
Flowchart of the Smart City Active Mobile Phone Intervention trial.

Figure 2 presents the flow of the participants. In total, 473 out of 4995 responded to the invitation letter and 254 completed baseline measures and were randomized (1:1). All accelerometer files went through an overall review to ascertain sufficient data recordings before randomization; however, when the accelerometer data were processed in detail after the study was completed, it was discovered that two participants in the control group did not fulfill the wear time criteria (≥600 minutes per day). Therefore, they were excluded from the analyses, and thus the final sample was n=252. No major differences were found between the 254 participants and the invited population-based sample regarding area of residence (city center or countryside) and age. However, participants were more often women, born in Sweden, and had a university degree compared with nonparticipants (Multimedia Appendix 2). There were no differences between the intervention and control group with respect to baseline characteristics (Table 1).

**Table 1:** Baseline characteristics of the participants in the Smart City Active Mobile Phone Intervention trial.

Variable		Control (n=125)	Intervention (n=127)
Age in years, mean (SD)		46.2 (11.0)	46.5 (11.0)
**Gender, n (%)**			
	Female	66 (52.8)	78 (61.4)
	Male	59 (47.2)	48 (37.8)
	Other	0 (0)	1 (0.8)
**Education, n (%)**			
	Primary	2 (1.6)	3 (2.4)
	Secondary	42 (33.6)	44 (34.6)
	Tertiary	81 (64.8)	80 (63.0)
Body mass index (kg/m^2^), mean (SD)		24.7 (3.1)	24.9 (4.0)
Sedentary^a,b^ (min/d), mean (SD)		485.4 (69.7)	477.9 (83.4)
Light activity^a,b^ (min/d), median (IQR)		310.8 (69.7)	320.6 (83.9)
Moderate to vigorous activity^a,b^ (min/d), mean (SD)		60.3 (26.0)	59.7 (27.6)
Moderate activity^a,b^ (min/d), mean (SD)		52.0 (22.0)	50.9 (23.2)
Vigorous activity^a,b^ (min/d), mean (SD)		8.2 (12.2)	8.8 (12.3)
**Active transportation^c^ (min/d), mean (SD)**		56.8 (26.5)	58.1 (28.1)
	Walking (min/d), mean (SD)	50.5 (25.8)	54.2 (27.9)
	Cycling (min/d), mean (SD)	6.3 (16.1)	3.9 (9.2)
General health^d^, mean (SD)		75.3 (17.7)	73.8 (17.1)
Perceived walkability^e^, mean (SD)		–0.2 (2.3)	0.2 (2.4)
**Attitude toward^f^, mean (SD)**			
	Walking	3.9 (0.6)	4.0 (0.5)
	Cycling	3.5 (0.8)	3.5 (0.8)
Counts per minute, mean (SD)^a^		389 (137.8)	390 (137.7)

^a^Measured by accelerometer.

^b^Wear time (days) for the accelerometer was 6.4 (SD 1.2) days (intervention) and 6.5 (SD 1.1) days (control). The corresponding wear time in minutes per day was 853 (SD 67) minutes (intervention) and 857 (SD 73) minutes (control).

^c^Measured by TravelVu smartphone app. Control (n=124) and intervention (n=126) due to missing data.

^d^Measured by RAND-36.

^e^Measured by means of Neighborhood Environment Walkability Scale.

^f^Measured by means of Transport and Physical Activity Questionnaire.

### Effectiveness of the Intervention

**Figure 3:**
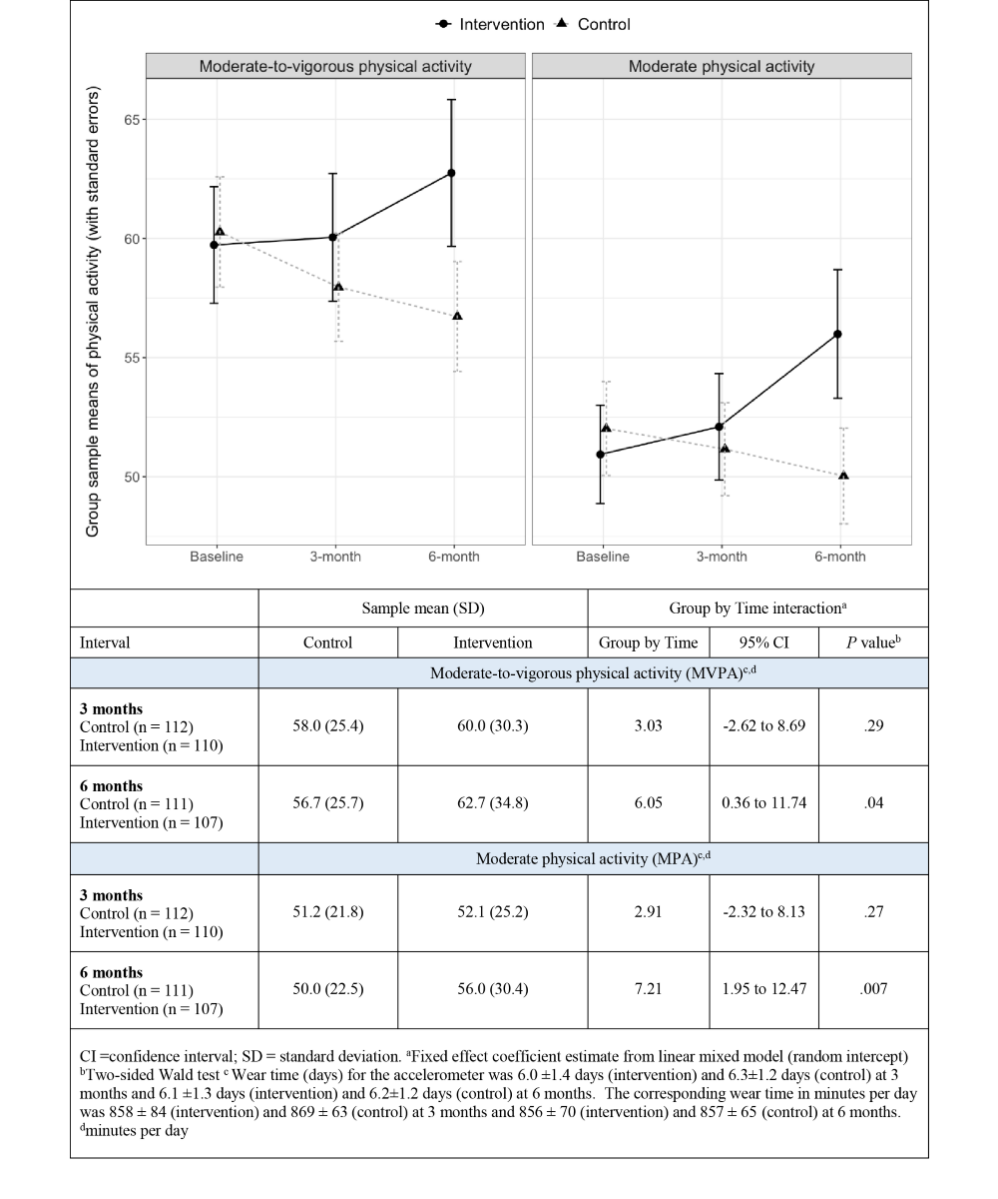
Intervention effect on moderate-to-vigorous physical activity and moderate physical activity at 3 and 6 months.

Results showed no statistically significant difference between groups on the primary outcome at 3 months (*P*=.29); however, at 6 months, the intervention group had a greater improvement in MVPA than the control group (6.05 minutes per day; 95% CI 0.36 to 11.74; *P*=.04). As shown in Figure 3, the difference in MVPA at 6 months was driven predominantly by changes in MPA (difference 7.21 minutes per day; 95% CI 1.95 to 12.47; *P*=.007). Sensitivity analysis (imputed data) revealed comparable results (Multimedia Appendix 3). There was an interaction between sex and the 6-month MVPA (group × time × gender coefficient estimate 14.7 minutes per day; 95% CI 3.2 to 26.1; *P*=.01), indicating the intervention was more effective in men than in women. There was no interaction effect for any of the other investigated covariates (ie, age, educational attainment, BMI, foreign background, season of randomization, perceived walkability index, attitude toward AT, or general health [results not shown]). AT (minutes per day) was statistically significantly associated with accelerometer MVPA (minutes per day; *r*=0.5; *P*<.001) at baseline, and this association remained similar at the two follow-ups. Pre-post differences of MVPA did not differ by app engagement (number of registered days in the app, goals set or achieved; results not shown). Table 2 presents the corresponding results for the secondary outcomes. No differences in the change of other secondary outcomes between follow-up and baseline were found between the groups. Correspondingly, no difference in the change of AT walking or AT cycling for transport was observed (results not shown). Finally, no statistically significant difference was found when contrasting light PA between groups.

**Figure 4:**
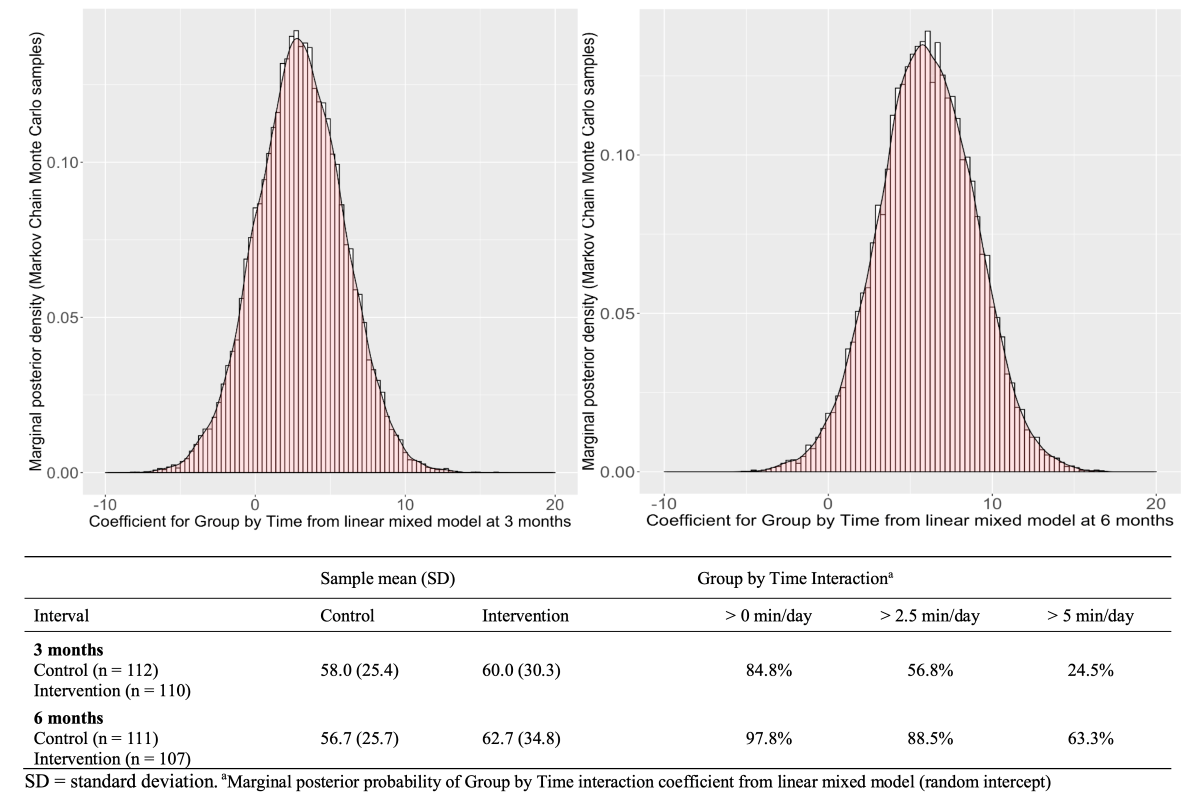
Bayesian analysis of the intervention effect on moderate-to-vigorous physical activity at 3 and 6 months.

Figure 4 reports the results from the Bayesian analyses for MVPA. The probability that the intervention group improved MVPA more than the control group (ie, had any effect on MVPA) was 84.8% at 3 months and 97.8% at 6 months. Furthermore, the probability that this improvement was more than 5 minutes per day was 63.3% at 6 months.

**Table 2:** The intervention effect on the secondary outcomes at 3 and 6 months.

Outcome			Sample, mean (SD)				Group × time interaction^a^				
			Control		Intervention		Group × time		95% CI^b^		*P* value^b^
**Active transportation^c^**											
	3 months^d^	58.0 (30.5)		58.6 (29.7)		0.96		–6.91 to 8.80		.81	
	6 months^e^	58.3 (29.0)		60.5 (32.4)		2.02		–6.39 to 10.4		.64	
**Attitude toward walking^f^**											
	3 months^g^	3.9 (0.6)		4.0 (0.6)		0.02		–0.11 to 0.14		.80	
	6 months^h^	4.0 (0.6)		4.0 (0.6)		–0.12		–0.25 to 0.01		.06	
**Attitude toward cycling^f^**											
	3 months^i^	3.6 (0.9)		3.5 (1.0)		–0.1		–0.25 to 0.06		.22	
	6 months^j^	3.7 (0.9)		3.5 (0.9)		–0.1		–0.26 to 0.05		.20	
**General health^k^**											
	3 months^l^	76.9 (18.5)		76.5 (16.7)		1.52		–1.69 to 4.74		.35	
	6 months^m^	75.3 (18.5)		77.2 (17.1)		3.17		–0.11 to 6.44		.06	

^a^Fixed effect coefficient estimate from linear mixed model (random intercept).

^b^Given by 2-sided Wald test.

^c^Measured by TravelVu (smartphone app).

^d^Control (n=102); intervention (n=106).

^e^Control (n=93); intervention (n=80).

^f^Measured by means of Transport and Physical Activity Questionnaire.

^g^Control (n=110); intervention (n=102).

^h^Control (n=107); intervention (n=95).

^i^Control (n=110); intervention (n=102).

^j^Control (n=107); intervention (n=95).

^k^Measured by RAND-36.

^l^Control (n=110); intervention (n=102).

^m^Control (n=107); intervention (n=95).

### App Engagement

Objective measures of engagement with the self-monitoring feature of AT in the standard TravelVu app for the two groups are shown in Table 3. For the intervention group, during the 3-month intervention period, 60.6% of participants (77/127) registered 57 days or more out of the 84 days in total, indicating high engagement. Also, as shown in Table 4, the goal setting function in the TravelVu Plus app was relatively well used with 58.2% of participants (74/127) in the intervention group setting weekly goals for 5 weeks or more and 46.4% (59/127) achieving those goals. App engagement decreased after the intervention period when participants only had access to the standard version (TravelVu); however, many participants in the intervention group continued to use the self-monitoring feature for the subsequent 3 months (Table 3).

In the control group (TravelVu), as seen in Table 3, 65.6% (82/125) continued to register days with AT in the app beyond the baseline assessment; however, the number of days (mean 39 [SD 35]) was fewer than for the intervention group (mean 53 [SD 32]; *P*=.01) during the first 3 months. During months 3 to 6 after baseline, the number of days were comparable (control: mean 34 [SD 34] days vs intervention: mean 30 [SD 32] days; *P*=.37).

**Table 3:** Objective measures of engagement with the self-monitoring feature of AT in the standard version of the app (TravelVu) in the intervention group (n=127) and control group (n=125).

Number of registered days in the app^a,b,c^	Intervention group		Control group	
	0 to 3 months, n (%)	3 to 6 months after intervention, n (%)	0 to 3 months, n (%)	3 to 6 months after intervention, n (%)
57-84	77 (60.6)	29 (22.8)	50 (40.0)	35 (28.0)
29-56	16 (12.6)	13 (10.2)	17 (13.6)	12 (9.6)
7-28	10 (7.9)	43 (33.9)	15 (12.0)	40 (32.0)
0-6	24 (18.9)	42 (33.1)	43 (34.4)	38 (30.4)

^a^A registered day is defined as a day that was reviewed and approved by participant as valid data regarding their travel behavior that day (ie, number of minutes spent walking, cycling).

^b^The maximum total number of days was 84 days since results are reported for 3 months or 12 weeks (ie, 0-3 months [intervention period] or 3-6 months [3-6 months after the intervention]).

^c^These categories correspond to <1 week, 1-4 weeks, 4-8 weeks and 8-12 weeks.

**Table 4:** Number of set and achieved goals in the TravelVu Plus app by the intervention group (n=127) during the 3-month intervention perioda.

Number of weekly goals in the app^b^	Set goals	Achieved goals
9-12	53 (41.7)	21 (16.5)
5-8	21 (16.5)	38 (29.9)
1-4	27 (21.3)	36 (28.3)
0	26 (20.5)	32 (25.5)

^a^The number of goals set and achieved provided in the table were extracted from the app (ie, objectively measured).

^b^The maximum number of goals is 12 since the intervention was 3 months (ie, 12 weeks).

## Discussion

### Principal Findings

This is the first RCT to determine the effect of a stand-alone behavior change program promoting AT delivered through a mobile phone app (ie, TravelVu Plus) on MVPA among adults. Access to the TravelVu Plus app did not lead to any statistically significant difference in MVPA at 3 months (primary outcome); however, relevant differences in MVPA were observed at 6 months. This difference was driven predominantly by an increase in MPA, which is reasonable since the intervention targeted cycling and walking. Bayesian analyses provided further support for an effect of the intervention.

### Comparison With Prior Work

To the best of our knowledge, only one previous study evaluated the effectiveness of an app designed to solely promote AT [12]. In that study, an increase in self-reported AT to campus among 563 university students was reported [12]; however, it was not possible to evaluate the effectiveness of the app alone since it was part of a multicomponent intervention. In our study, there are several possible explanations for why there was an intervention effect at 6 months but not at 3 months. First, the intervention group may have needed a longer time period to achieve a behavior change (ie, increase their AT). Thus, even though the intervention group was less engaged in the app after 3 months when the behavior change features were disabled, it may be speculated that they had found strategies during the intervention and then applied those strategies to increase AT (detectable at 6 months). Second, in accordance with the predetermined study protocol, both groups had access to the TravelVu app in order to objectively assess AT during 7 days at baseline and the two follow-ups. Although the control group did not receive the 3-month enhanced app features, it is possible that using the standard app, anchored in self-monitoring [40], made them aware of their PA pattern and thus influenced them to engage in more PA in the beginning of the study period and potentially diluted the intervention effect at 3 months. Future studies should investigate whether use of the TravelVu Plus app would result in a larger difference when compared with a control group with no app use.

It is also relevant to compare our results with other app-based PA interventions. Systematic reviews and meta-analyses have concluded that mobile phone–based interventions may have small to moderate effect sizes when it comes to improving PA in free-living adults [41-44]. A recent study showed a mean increase in daily step count of between 226 and 319 steps associated with four different types of mobile phone interventions, which equated to around 5% of the mean daily step count for American adults (4700 steps per day) [45]. Thus, our findings are comparable to previous app-based PA interventions and extend available literature since we only targeted AT as a PA behavior.

To the best of our knowledge, this is the first trial that evaluated objectively assessed AT via GPS monitoring instead of using self-report as a secondary outcome. This approach relied on participants’ approval of trips taken and making corrections to these as necessary (ie, ensuring the trip was undertaken as indicated in the app). Notwithstanding, participants in the intervention group approved 71% of their travel trips (60 out of 84 days), indicating high use of this feature to self-monitor their AT. Furthermore, although the control group only had access to the standard version of the app, they used it for an average of 39 days, which supports that they also appreciated monitoring AT. Still, given the required level of interaction, only 65% of participants provided complete data on AT for baseline and the two follow-up assessments. This loss of data likely contributed to not being able to detect an effect on AT, and thus findings for AT as outcome should be interpreted with caution. Our original preference was to passively record AT by a mobile app for the assessment period only (it would be shut off automatically between the baseline and follow-up periods); however, this was not technically feasible at that time. Future studies should consider how to address these issues to optimize objectively assessed AT.

### Strengths, Limitations, and Generalizability

Strengths of the SCAMPI trial include the RCT design, as well as the objectively measured primary outcome (MVPA). Previous studies on AT and PA are mostly observational, and systematic reviews have called for well-designed interventions targeting AT [8,11,46]. The included Bayesian analyses further strengthen the hypothesis that the TravelVu Plus app helped individuals increase their MVPA.

The main limitation of this study is that the primary outcome (MVPA) was assessed by accelerometry which does not capture cycling behavior; however, this choice was carefully considered when designing the study. Notwithstanding its limitations, an objective measure of PA was considered preferable to self-report as there is no well-established and objective measure to assess AT (walking and cycling). We also complemented the accelerometer data with GPS data to assess AT (walking and cycling) as the secondary outcome. The GPS data indicated that in our study approximately 90% of AT was walking, which was captured with accelerometry. Thus, it is reasonable to conclude that most likely our effects on MVPA are slightly underestimated due to the fact that the accelerometer could not capture cycling. Also, we cannot exclude that some AT could be light PA instead of MVPA; however, no difference was found when contrasting light PA between groups. Another important limitation to consider is seasonality. Sweden has a relatively cold climate with a winter period between December and February; however, the autumn and spring months can also be quite cold and snowy, which does not facilitate AT. As described above, we recruited in two waves (early autumn and early spring) in order to spread the intervention period out throughout different seasons, and we did not find any evidence that the effectiveness of the intervention differed upon which season participants entered the trial. Finally, other limitations of this trial include that automated feedback messages were used instead of personalized, that the review and correction of the automatically captured AT was considered time-consuming by some participants, and that the app consumed quite a lot of battery power (as indicated in the postintervention qualitative interviews, reported separately).

To minimize the risk for selection bias, recruitment was population-based with a sample randomly drawn by Statistics Sweden. Nevertheless, as is common in research, the willingness to participate was higher among women and well-educated people. However, the effect was not moderated by baseline educational attainment. Furthermore, both groups had on average 60 minutes MVPA at baseline. No comparable population-based data regarding MVPA exists in Sweden, and comparisons between studies are difficult due to different protocols and accelerometer cut points; however, previous studies in Swedish adult populations have shown approximately 35 minutes of MVPA per day [47-49]. Thus, although a random sample was drawn, we cannot exclude that people who signed up had a relatively physically active lifestyle, and the potential of the TravelVU Plus app to promote AT in more sedentary populations should be explored. Also, we cannot generalize our results to people who recently migrated to Sweden since the app is currently only available in Swedish. Finally, it is relevant to note that Stockholm is a city amenable to AT (walking and cycling); however, many cities and suburbs across the world are not, which may limit generalizability to other types of cities as well as limit the reach of interventions targeting only AT. Thus, future studies should evaluate the potential of apps to promote AT similar to the TravelVU Plus app in other types of cities and contexts.

### Implications and Future Research

The SCAMPI trial provides several important findings and insights for future interventions targeting AT. First, objective measures showed that the TravelVu Plus app was used frequently for 3 months, indicating its potential for promoting AT. This finding was supported by the in-depth interviews with a subsample (reported separately). Furthermore, this qualitative data suggested the need for improvements such as personalized messages and an improved function for registrations, preferably automatized if possible, for correction of travel trips in the app. These improvements may enhance the effectiveness of the app. Second, we were able to observe a 6-minute difference in MVPA in the intervention group compared with the control group at 6 months. The observed effect is modest; however, considering that 6 minutes per day adds up to 42 minutes per week which corresponds to nearly 30% of the recommendation (150 minutes MVPA per week) [50] and it was achieved through a low-cost and scalable intervention, this finding is important. In this context it is also important to highlight that, due to the 24-hour continuum, adding time in AT is a reduction of time spent in other activities—in this case most likely a reduction in sedentary time (replacing motor transport) which also has positive health implications. Previous data indicate that Swedish adults spend as much as 60% of their total time as sedentary [47] and also that very small substitutions of sedentary time for MPA have shown decreased risks for metabolic syndrome [51,52]. Also, it may be speculated that the observed effect might have been larger if the control group did not have access to the app for the measurement of AT. Thus, it is reasonable to conclude that our findings together with the high app engagement motivates further investigation of the potential of the TravelVu Plus app to promote AT.

### Conclusions

We observed no effect on MVPA immediately after the intervention period (at 3 months); however, there was a delayed effect on MVPA (6 minutes per day) at 6 months, which corresponds to almost 30% of the weekly MVPA recommendation. Our findings coupled with the high engagement in the app suggest that a behavior change program promoting AT delivered through an app may have a relevant effect on PA, motivating further research on mHealth AT interventions.

## Appendix

Multimedia Appendix 1 Screenshots from the app showing examples of messages, weekly summary of travel and number of achieved weekly goals.

Multimedia Appendix 2 Supplementary Table 1.

Multimedia Appendix 3 Supplementary Table 2.

Multimedia Appendix 4 CONSORT-EHEALTH checklist (V 1.6.1).

## Abbreviations

AT: active transportation
CONSORT-EHEALTH: Consolidated Standards of Reporting Trials of Electronic and Mobile Health Applications and Online Telehealth
mHealth: mobile health
MPA: moderate physical activity
MVPA: moderate-to-vigorous physical activity
PA: physical activity
RCT: randomized controlled trial
SCAMPI: Smart City Active Mobile Phone Intervention
VM: vector magnitude
