# Supplementary figures and images for: Effectiveness of a 3-Month Mobile Phone–Based Behavior Change Program on Active Transportation and Physical Activity in Adults: Randomized Controlled Trial
Source: JMIR Mhealth Uhealth. 2020 Jun 8;8(6):e18531. doi: 10.2196/18531 (PMC7308910; doi:10.2196/18531)

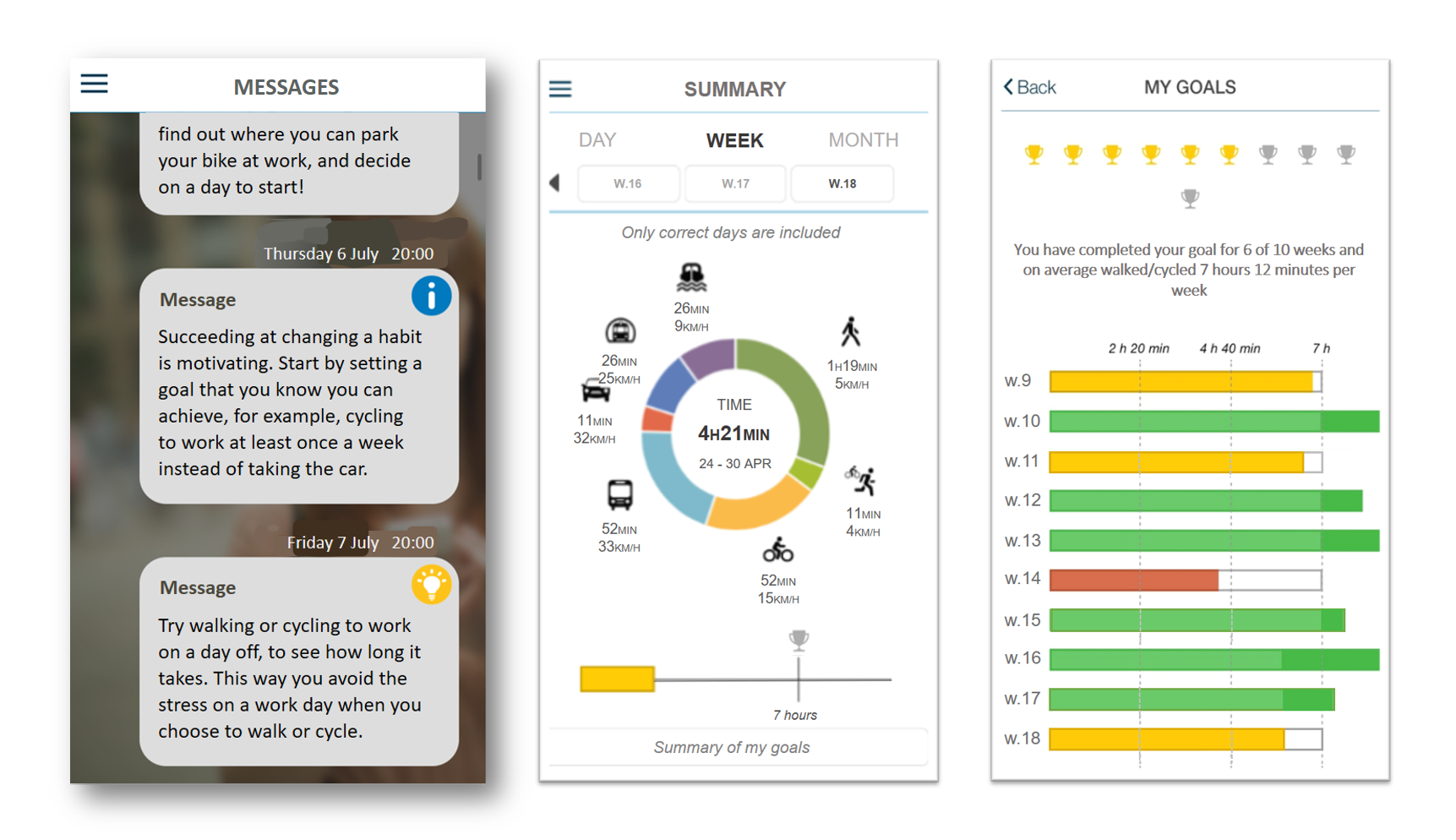

Supplement: Multimedia Appendix 1 [file mhealth_v8i6e18531_app1.png]
