# Supplementary material for: Effectiveness of a 3-Month Mobile Phone–Based Behavior Change Program on Active Transportation and Physical Activity in Adults: Randomized Controlled Trial
Source: JMIR Mhealth Uhealth. 2020 Jun 8;8(6):e18531. doi: 10.2196/18531 (PMC7308910; doi:10.2196/18531)
Supplement: Multimedia Appendix 2 [file mhealth_v8i6e18531_app2.pdf]

Multimedia Appendix 1

Supplementary Table 1. Characteristics of the total population that received an enquiry letter about the study (adults aged 18-65 living in Stockholm County) and the SCAMPI participants.

	**Total population (n=4995)**		**SCAMPI participants (n=254)**	
	n	%	n	%
**Gender**				
Men	2561	51.3	108	42.5
Women	2434	48.7	146	57.5
**Age**				
20-29	1094	21.9	23	9.1
30-39	1192	23.9	52	20.5
40-49	1227	24.6	79	31.1
50-59	1035	20.7	71	28.0
60-69	447	8.9	29	11.4
**Country of birth**				
Sweden	3452	69.1	218	85.8
Other	1543	30.9	36	14.2
**Income**				
None	516	10.3	5	2.0
Very low	828	16.6	18	7.1
Low	441	8.8	9	3.5
Middle	582	11.7	17	6.7
Middle/High	887	17.8	55	21.7
High	1741	34.9	150	59.1
**Education**				
Primary school	585	11.7	6	2.4
High school	1889	37.8	73	28.7
University degree, < 3y	790	15.8	49	19.3
University degree, > 3y	1429	28.6	126	49.6
**Residential area**				
C - municipality or city center	4609	92.3	235	92.5
B - municipality or city	102	2.1	6	2.4
A - countryside	197	3.9	8	3.2
Unknown	87	1.7	5	1.9

Note: Annual income categories: Very low, 1 - 124 999 Swedish crowns (SEK); Low, 125 000 -199 999 SEK; Middle, 200 000 -279 999 SEK; Middle/High, 280 000 -369 999 SEK; High, >370 000 – SEK

Residential area categorized according to Statistics of Sweden, A: largest part outside of larger population concentrations; B: largest part within population concentrations; C: largest part within a municipality or city center (https://www.scb.se/hitta-statistik/regional-statistik-och-kartor/regionala-indelningar/deso---demografiska-statistikomraden/)
