# Supplementary material for: Effectiveness of a 3-Month Mobile Phone–Based Behavior Change Program on Active Transportation and Physical Activity in Adults: Randomized Controlled Trial
Source: JMIR Mhealth Uhealth. 2020 Jun 8;8(6):e18531. doi: 10.2196/18531 (PMC7308910; doi:10.2196/18531)
Supplement: Multimedia Appendix 3 [file mhealth_v8i6e18531_app3.pdf]

Multimedia Appendix 2

Supplementary Table 2. The intervention effect on the primary outcome derived from the multiple imputation analyses[a].

	Moderate-to-vigorous physical activity				
	**Sample mean (SD)**		**Group by Time Interaction[b]**		
**Interval**	**Control**	**Intervention**	**Group by Time**	**95% CI**	***P* value[c]**
**3 months**					
Control (n = 125)	58.1 (25.7)	60.9 (30.2)	3.26	-2.49 to 9.01	.27
Intervention (n = 127)					
**6 months**					
Control (n = 125)	57.6 (26.4)	62.9 (33.6)	5.69	-0.03 to 11.42	.051
Intervention (n = 127)					

CI = confidence intervals; SD = standard deviation

[a]Differences between those who did and did not complete follow-up was explored through regression models using baseline characteristics as covariates. Four baseline characteristics were found to be associated with completers at 3 months: a greater number of minutes spent on light and sedentary activity, a higher BMI, and a more positive attitude towards cycling. No association between baseline characteristics and 6 month completers were found, however there was an association between 3 and 6 month completers. Overall, this is evidence against missing complete at random, however, it was decided that the four baseline characteristics could reasonably explain why the data was missing, i.e., those who have less to gain from the use of the intervention were less willing to complete follow-up, and therefore primary analyses were redone using imputed data.

[b]Fixed effect coefficient estimate from linear mixed model (random intercept)

[c]Two-sided Wald test
